# Epigenetic aspects of telocytes/cordocytes: jacks of all trades, masters of most

**DOI:** 10.3389/fncel.2014.00032

**Published:** 2014-02-10

**Authors:** Lawrence Edelstein, John Smythies

**Affiliations:** ^1^Medimark CorporationDel Mar, CA, USA; ^2^Department of Psychology, Center for Brain and CognitionUCSD, San Diego, CA, USA

**Keywords:** telocytes, cordocytes, exosomes, tissue repair, electrical rhythms, stem cells

## Introduction

In a recent review (Smythies and Edelstein, 2013b) we described the structure and function of a new form of interstitial cell found in most mammalian organs, the telocyte/cordocyte (T/C) co-discovered and ascribed accordingly by teams led by Laurentiu Popescu (Popescu et al., 2005; “telocytes”) and Viorel Pais (Danaila and Pais, 2011; Pais et al., 2012, 2013a,b; “cordocytes”). The problem of nomenclature is further complicated by the persistent use of an old name—“interstitial cells of Cajal” or ICCs (e.g., Padhi et al., 2013; Tanahashi et al., 2013). T/Cs make synaptic contacts of various kinds (including puncta adhaerens and gap junctions) with a broad variety of cells and tissue (including blood vessels, nerve fibers, fibroblasts, muscle cells, immune cells, and glandular cells, as well as other T/Cs). The present consensus is that T/Cs are capable of forming an extensive intercellular information transmission and executive system that may utilize electric currents, small molecules, exosomes—and possibly electrical events within the cytoskeleton—to modulate homeostasis, stem cell activity, tissue repair, peristalsis, anticancer activity, and other complex functions in many organs. We herein present a more comprehensive hypothesis of the molecular and cellular bases of the function of T/Cs with a focus on mechanisms for tissue repair.

## The facts

Recently published studies demonstrate that cordocytes (T/Cs) are highly dynamic cells capable of rapidly changing their shape, position, and connections (Danaila and Pais, 2011; Pais et al., 2012, 2013a). These workers also report them as being extensively distributed in the brain in relation to blood vessels, as well as the pia mater and choroid plexus. T/Cs have been shown to rapidly migrate to an injured area via the rapid growth of their long, sinuous processes. In many cases these processes envelop the damaged tissue, with a particular emphasis on repair and abating hemorrhages. As well, (Pais et al., 2013a,b) observed T/C processes closely enveloping tumor tissue in the brain, suggestive of some form of antitumor activity. The authors go on to say (Pais et al., 2013a):

“Under their strict surveillance all cellular movements are controlled, and under their efficient protection all vessels are surrounded by cordocytes, these cells primarily acting against erythrocyte extravasation [i.e., bleeding]… Cordocytes have a fundamental role in cooperation with stem cells for the generation of new cells during regeneration and repair events throughout the brain. These special interstitial cells coordinate and direct stem cells to damaged areas.”

Pais (2013) concludes “In the last instance, the brain performance improves if its microenvironment is maintained under appropriate conditions, for which cordocytes are responsible.”

## Comments

Further analysis of the extant data leads us to consider: (1) an addition to our present hypothesis (Smythies and Edelstein, 2013b), and (2) a further development of the hypothesis itself.

(1) The description by Pais et al. (2012, 2013a) of the dynamic and motile behavior of cordocytes must be reconciled with the earlier perception of T/Cs being fixed in place or static. In our previous paper (Smythies and Edelstein, 2013b) we drew attention to the possibility that telocytes might convey information by a process akin to the classical model by which otherwise static neurons transmit information, namely, by sending electrical and/or chemical messages along their lengths. This now needs to be complemented by an additional mechanism, whereby the cell literally moves itself (and its cooperative stem cells) by extending its protrusions (telopodes) to perform information transmittal and executive functions related to damage control and repair. The arrival of the priming signal from the extravasating vessel (or cancer cell) which the T/C is monitoring, may trigger the intracellular release of calcium (Yamashita, 2010). This would then depolymerize the actin skeleton (Rosenmund and Westbrook, 1993) in order that the normally stationary and elongated telopode can assume a mobile form. However, with that said, this addition may not lead us too far from the current model, since it is now known that, although neurons themselves do not carry messages by migrating bodily, nevertheless, they continually alter the spatio-temporal pattern of their synaptic connections with other neurons by pruning old synapses and sprouting new ones (Smythies and Edelstein, 2013a).

(2) In the previous formulation of our hypothesis we dealt with T/Cs as single information conducting and processing units, each with numerous and sundry anatomical inputs and outputs with different functions, integrated over vast distances via flimsy conduits or telopodes. This led to problems concerned with the amount of information that could be transmitted in such a manner, and the appreciable length of time required before repair critical mechanisms could be activated. Furthermore, we were unable to provide a satisfactory account of the role of exosomes in this system. We have now come to the realization that one simple change in the system obviates these sticking points. Our amended hypothesis is that the T/C is not a single and otherwise independent information processing unit. Rather, it is comprised of a large number of local independent processing units in which exosomes play a key role. This system operates, we suggest, as follows:

During the process of tissue differentiation and organ development T/Cs randomly infiltrate the tissue, making multiple synapses on a variety of specific target cells (STC); the two commence to exchange exosomes. The exosomes from the STC establish a molecular profile/mechanism in an immediately adjacent section of the T/C (e.g., via exosome uptake sites or puncta adhaerens), specific for the processing of signals from that type of STC. Subsequently, when that STC is damaged, it emits molecular signals detailing the components of such (e.g., hemoglobin in the case of a damaged blood vessel). Furthermore injured cells rapidly change the specific RNA content of their emitted exosomes (D'Alessandra et al., 2010; Loyer et al., 2014). Therefore the signaling molecules we postulate to be emitted by STCs may include cell- and injury-specific miRNAs. The exosomes initially exported in the direction STC→T/C carry epigenetic material that organizes and promotes the development of the molecular mechanisms in the T/C that (a) would be activated by this specific signal and (b) would then mobilize the molecular and cellular machinery necessary to repair the damage. This might be accomplished by contacting and activating local stem cells to multiply and develop the specific features needed to repair the damage in that particular type of cell and injury. Inherent to this process would be the rapid initiation of a massive increase in the production of plasma membrane which, for example, might be needed to enrobe a leaking blood vessel. The T/Cs would otherwise remain relatively static, and the epigenetically-evoked machinery would lie dormant, until such time that an STC signals that it is damaged. At that time, the locale-specific signaling molecule would be transported to the T/C, either directly or via exosomes, and the repair machinery would be activated immediately. This mechanism would reduce the need for long distance and decidedly slow information transfer in the T/C to a minimum. This mechanism is reminiscent of the way in which ribosomes are packed around the base of dendritic spines in the brain (Dynes and Steward, 2012; Jasinska et al., 2013), allowing for much more rapid new protein synthesis in response to stimulation than if the signal had to be routed to the cell nucleus itself to accomplish the task. There is a precedent for this mechanism in the neural synapse. Smalheiser (2007) has provided evidence that exosomes which have budded-off from the postsynaptic neuron can carry epigenetic molecules that modulate the function of the presynaptic neuron. In essence, this is the mechanism that we propose, only operating at the T/C-STC synapse.

Specific signals from damaged tissue could also evoke motor processes in the cytoskeleton, possibly via calcium flows and the electrical signals in the cytoskeleton that we detailed in our previous paper (Smythies and Edelstein, 2013b), such that the cell moves itself and/or its protrusions to the site of injury. At that point, more distant T/Cs could be recruited by direct signals transmitted via the electrical junction-linked network that joins T/Cs. This process may run parallel to the intracellular processes which we also described in that paper Smythies and Edelstein (2013b). In this way, T/Cs could efficiently and rapidly congregate to the lesioned site, as observed by Pais et al. (2012, 2013a).

During the process of sealing-off a bleed, or surrounding a cancer cell (Pais et al., 2013a), the T/C has to supply a large quantity of local membrane to enrobe the area of concern. How does it do this? We suggest two possible mechanisms:

1. The activated T/C could stimulate its attached stem cell(s) to differentiate into the cells needed to produce this membrane and repair the damage.
2. Electron micrographs reveal that telopodes can fold upon themselves in a beta-sheet-like conformation, thus providing a large quantity of locally redundant membrane akin to a hawser coiled on a ship's deck (see Figure 4 in Cretoiu et al., 2012). This could also be used to provide extra membrane for protecting the repair.

Yet the function of the T/C as an “intelligent bandage” cannot be the whole story. Telocytes are reported to contact many other cell types and structures besides blood vessels and cancer cells. For example, they appear to play a role in modulating contractile activity in smooth muscle. Cordocytes are reported as being capable of spontaneously initiating rhythmic electrical activity (Danaila and Pais, 2011). These rhythms can be modulated by the stimulation of enteric nerves (Zhu et al., 2013); this process involves calcium transients. There is also evidence that ICCs may mediate cholinergic neuromuscular transmission in ileal smooth muscle (Tanahashi et al., 2013).

Thus, in the case where the STC is a smooth muscle cell, and the T/C modulates its frequency of contraction as in peristalsis, the machinery set-up by the STC-originated exosomes in the T/C might relate to the generation of slow wave potentials exported from the T/C to the STC. Similar mechanisms might be involved in the case of the other STCs reported. For example, when the STC is a macrophage or lymphocyte, the machinery set-up by exosome transfer to the T/C would be specific to immune reactions. In this way, a very complex job is done by an array of very simple mechanisms. Furthermore, specification of the various roles of different parts of the T/C does not require any elaborate information-carrying mechanism within the T/C itself. All of this is supplied automatically from local STCs via exosomes.

## Conclusion

Very recent data suggests that the T/C may have even more remarkable properties and functions than we had previously suggested and considered. Firstly, in addition to its role in static information transfer and control functions mediated by intracellular electrical and chemical messages (which themselves integrate the function of many cell types in multiple organs), it may also have dynamic properties which enable it to literally move itself to the scene of an accident or insult and render first aid. Secondly, we propose a much improved mechanism for T/C-mediated activation of repair and cognate mechanisms, in which exosomes bearing differential epigenetic loads play a key role, based on the establishment of autonomous local units for tissue repair and modulation. In this way a purely random collection of cell-cell interactions yields a mechanism of extreme finesse and complexity. It seems remarkable indeed that so simple a cell can perform so many varied functions. In this regard, its properties seem not unlike that of the claustrum (Smythies et al., 2014). If we are right, both are able to integrate multiple functions by means of an array of differently tuned yet simple mechanisms.
